# B Cells Are Multifunctional Players in Multiple Sclerosis Pathogenesis: Insights from Therapeutic Interventions

**DOI:** 10.3389/fimmu.2015.00642

**Published:** 2015-12-21

**Authors:** Nele Claes, Judith Fraussen, Piet Stinissen, Raymond Hupperts, Veerle Somers

**Affiliations:** ^1^Hasselt University, Biomedical Research Institute and Transnationale Universiteit Limburg, School of Life Sciences, Diepenbeek, Belgium; ^2^Department of Neuroscience, School of Mental Health and Neuroscience, Maastricht University, Maastricht, Netherlands; ^3^Department of Neurology, Academic MS Center Limburg, Zuyderland Medisch Centrum, Sittard, Netherlands

**Keywords:** multiple sclerosis, B cell subtypes, therapy, antibodies, cytokines, costimulation, antigen presentation

## Abstract

Multiple sclerosis (MS) is a severe disease of the central nervous system (CNS) characterized by autoimmune inflammation and neurodegeneration. Historically, damage to the CNS was thought to be mediated predominantly by activated pro-inflammatory T cells. B cell involvement in the pathogenesis of MS was solely attributed to autoantibody production. The first clues for the involvement of antibody-independent B cell functions in MS pathology came from positive results in clinical trials of the B cell-depleting treatment rituximab in patients with relapsing-remitting (RR) MS. The survival of antibody-secreting plasma cells and decrease in T cell numbers indicated the importance of other B cell functions in MS such as antigen presentation, costimulation, and cytokine production. Rituximab provided us with an example of how clinical trials can lead to new research opportunities concerning B cell biology. Moreover, analysis of the antibody-independent B cell functions in MS has gained interest since these trials. Limited information is present on the effects of current immunomodulatory therapies on B cell functions, although effects of both first-line (interferon, glatiramer acetate, dimethyl fumarate, and teriflunomide), second-line (fingolimod, natalizumab), and even third-line (monoclonal antibody therapies) treatments on B cell subtype distribution, expression of functional surface markers, and secretion of different cytokines by B cells have been studied to some extent. In this review, we summarize the effects of different MS-related treatments on B cell functions that have been described up to now in order to find new research opportunities and contribute to the understanding of the pathogenesis of MS.

## Introduction

Multiple sclerosis (MS) is a chronic inflammatory disease of the central nervous system (CNS), characterized by demyelination in white and gray matter regions, axonal degeneration, and gliosis (1). MS is the most common chronic neurological disease in young adults affecting more women than men (three to one) with an incidence of 7/100,000 and a prevalence of 120/100,000 in Northern Europe (1). The diagnosis of MS is mostly preceded by a clinically isolated syndrome (CIS), which is the first clinical manifestation of a demyelinating disease that has not met the criteria of MS yet (2). Different clinical subtypes of MS are described. About 80% of the patients present with relapsing-remitting (RR) MS, which is characterized by disease exacerbations with periods of functional improvement (3). Over time, about 60% of the RRMS patients develop secondary progressive (SP) MS (4). About 10–20% of MS patients show progressive accumulation of disability from onset, referred to as primary progressive (PP) MS (2). According to the revised definitions of MS, above mentioned MS subtypes can present themselves in an inactive and active form (2). The underlying process of disease progression is not completely understood (5). Most MS therapies are primarily designed as treatment for RRMS patients, where there is marked inflammation.

Current data support the conceptual idea of MS as a complex heterogeneous disease caused by interactions between the environment, genetic susceptibility, and a dysbalanced immune system (6–8). Traditionally, T cells were considered as critical immune components required for the induction of MS pathogenesis. Recently, compelling evidence is present highlighting B cells as central components of the disease as well (9, 10). Autoreactive T cells are activated in the periphery most likely via molecular mimicry or bystander activation and home through a disrupted blood–brain barrier (BBB) to the CNS, where they are reactivated by antigen-presenting cells. This triggers the production of different mediators, such as chemokines and cytokines, by T cells, microglia, and other cells of the CNS. This will in turn initiate the recruitment of other inflammatory cells, including B cells and macrophages. B cells have the ability to cross the BBB and undergo stimulation, antigen-driven affinity maturation, and clonal expansion (11). The inflammatory reaction of T, B, and other immune cells leads to demyelinated lesions throughout the CNS (3).

As B cell involvement in MS has become more evident in recent years, more data have been collected concerning the effects of B cells in MS pathogenesis. Proof of B cell involvement in MS is described thoroughly further on in the review. Both B cell subtype distribution and B cell effector functions are important contributors to the disease. These processes are first described in more detail in order to fully understand how these processes are affected in MS patients and modulated by different MS treatments.

### B Cell Subtype Distribution in MS

**Figure 1:**
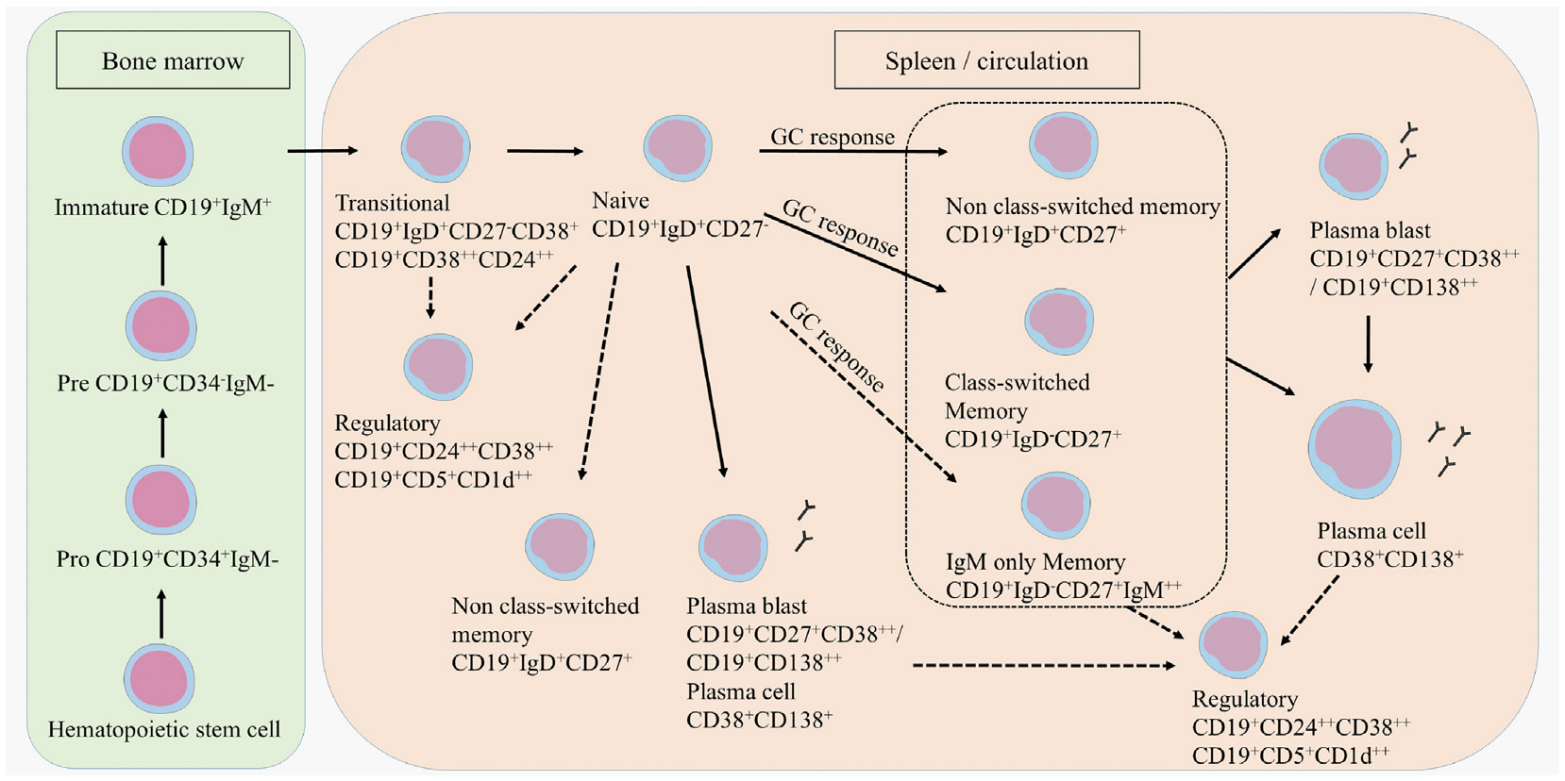
**B cell development**. B cells develop in the bone marrow and enter the circulation as transitional B cells. B cells remain naive until they encounter an antigen after which they differentiate into plasma blasts, short-lived plasma cells, or further mature into class-switched or non-class-switched memory B cells in a GC response. However, non-class-switched memory B cells can also be formed independent of a GC. A proportion of the memory B cells further develops into plasma blasts and/or plasma cells. Regulatory B cells are characterized within the transitional, naive, memory, and plasma blast or plasma cell population. Potential developmental routes are indicated with the dotted lines.

B cell development starts in the bone marrow where a hematopoietic stem cell evolves into an immature CD19^+^ B cell (Figure 1) (12). Transitional B cells (CD19^+^CD38^++^CD24^++^ or CD19^+^CD27^−^IgD^+^CD38^+^) enter the circulation and mature into naive B cells (CD19^+^IgD^+^CD27^−^). Upon antigen recognition, naive B cells proliferate into short-lived plasma blasts (CD19^+^CD138^++^ or CD19^+^CD27^+^CD38^++^) or plasma cells (CD38^+^CD138^+^) that produce low-affinity antibodies for a few days or further mature into memory B cells (CD19^+^CD27^+^) in a germinal center (GC) reaction. A proportion of memory B cells remains non-class-switched memory cells (CD19^+^IgD^+^CD27^+^), while others lose their immunoglobulin (Ig)D expression following isotype switching (CD19^+^IgD^−^CD27^+^). This classically results in the surface expression of IgG, IgA, or IgE, although a small proportion of memory B cells preserve IgM surface expression, namely IgM only memory B cells (CD19^+^IgD^−^CD27^+^IgM^++^) (13–17). A proportion of the memory B cells further matures into plasma blasts and long-lived plasma cells.

T cell subtypes important for providing help in the GC reactions are follicular helper T cells (TFH), follicular regulatory T cells (TFR), but also Th17 cells that can all induce or regulate GC formation and isotype switching (18–21). Regulatory B cells (Bregs) have been identified more recently by their function in immune regulation via the production of IL-10 (22, 23). Bregs could be enriched from transitional B cells, CD27^+^ memory B cells and plasma cells. Surface markers to characterize Bregs are still not clearly defined, although in humans CD24, CD38, CD5, and CD1d are mostly used (24–26).

Compositional changes of B cell subtypes in the peripheral blood (PB) are evidenced, shifting the balance toward more pro-inflammatory responses and less regulation. It is thought that memory B cells, plasma blasts and plasma cells preferentially cross the disrupted BBB and migrate into the CNS of MS patients, where they dominate the B cell pool and exert different effector functions (11, 27–35). During MS relapses, the percentage of PB memory B cells is increased (36). As TFH and TFR cells contribute to a normal GC response wherein potential autoantibodies are eliminated, the altered TFH and TFR function observed in MS patients can result in an inadequate GC response and the production of autoantibodies in the PB (18, 19).

In contrast to an increased percentage of memory B cells in PB, the proportion of Bregs was decreased in MS patients, while unchanged compared to healthy donors in other studies (37–40). Breg function was shown to be preserved as no differences were observed between MS patients and healthy donors in the ability of Bregs to inhibit proliferation of CD4^+^CD25^−^ T responder cells (40).

### B Cell Effector Functions

**Figure 2:**
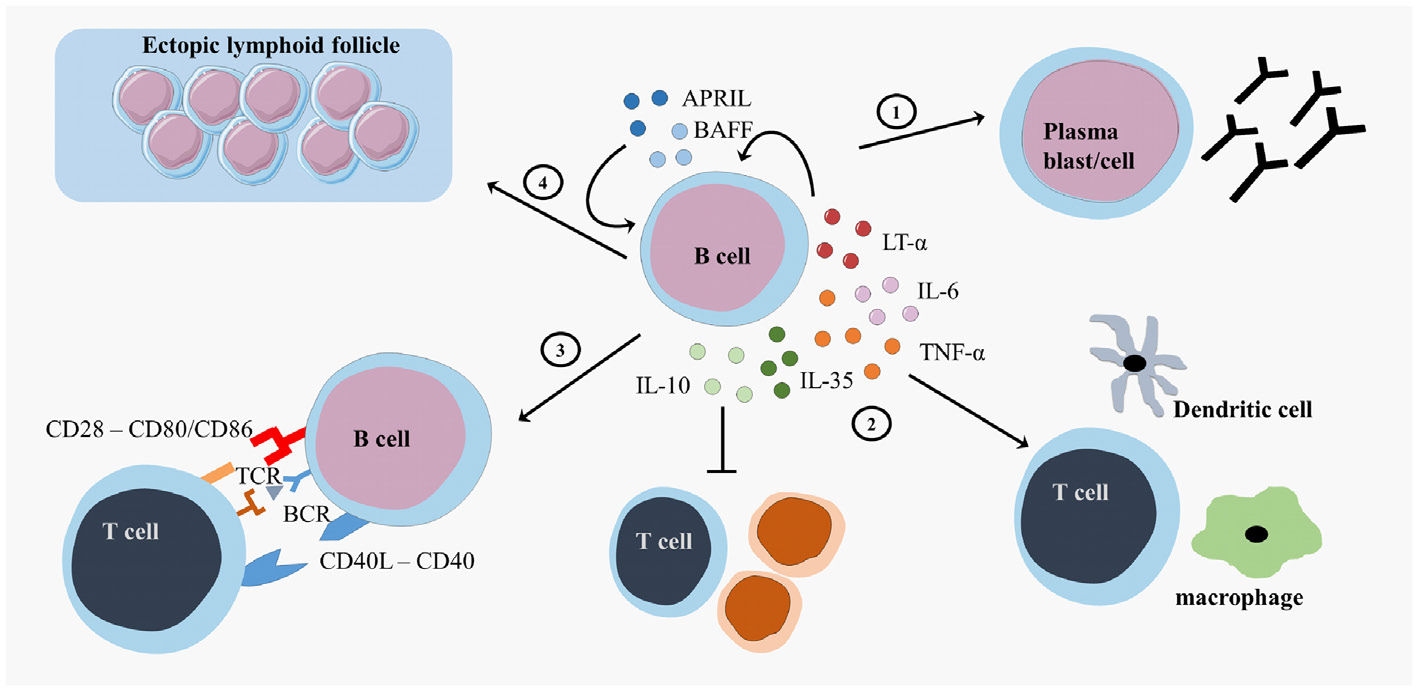
**B cell effector functions**. B cells exert different effector functions. B cells evolve into plasma blasts or plasma cells and produce antibodies (1). B cells produce different pro-inflammatory cytokines (lymphotoxin (LT)-α, tumor necrosis factor (TNF)-α, interleukin (IL)-6 or regulatory cytokines (IL-10, IL-35)) that influence other immune cells (2). B cells present antigens to T cells and provide costimulatory signals in order to induce appropriate T cell responses (3). B cells form ectopic lymphoid follicles that support the inflammatory responses (4). CD, cluster of differentiation; CD40L, CD40 ligand; APRIL, a proliferation-inducing ligand; BAFF, B cell activating factor; TCR, T cell receptor; BCR, B cell receptor.

B cells exert multiple effector functions, which are relevant to the pathogenesis and therapy of MS (9). First, B cells differentiate into antibody-secreting plasma blasts and plasma cells and produce antigen-specific antibodies (Figure 2). IgG from MS patients caused demyelination and axonal damage in a complement-dependent manner when using both *in vivo* and *in vitro* models (41, 42). Plasmapheresis and immunoadsorption in order to remove antibodies and complement factors already showed promising results as treatment for MS patients with steroid-resistant relapses (43, 44). In MS, different antibody targets have been described, including myelin basic protein (MBP), myelin oligodendrocyte glycoprotein (MOG), neurofilament, sperm-associated antigen 16 (SPAG16), coronin-1a, heat shock proteins, and other components of the CNS, emphasizing the diversity and complexity of the antibody response (45–54). An extensive review on different antibody targets is found in Ref. (45).

Second, B cells form GC-like structures, ectopic lymphoid follicles, outside of secondary lymphoid organs at sites of inflammation (Figure 2). These follicles harbor a local source of class-switched Igs that contribute to the immune response and are detected as oligoclonal bands (OCB) in the cerebrospinal fluid (CSF) of MS patients (55–57). These OCB in the CSF of MS patients were one of the first findings for B cell involvement in MS (58, 59). Intrathecal B cells are the local source for these OCB in the CSF, contributing to inflammation, and the destruction of the myelin sheet in the CNS (60). B cells migrate to the CNS using surface markers such as C–X–C motif receptor (CXCR)3, CXCR5, and CC chemokine receptor (CCR)5. The CNS has a fostering environment in which the production of CXCL10 and CXCL13 attracts B cells (61). In the meninges of MS patients, these migrated B cells form ectopic GC structures (57).

Third, B cells serve as highly effective and selective antigen-presenting cells leading to optimal antigen-specific T cell expansion, memory formation, and cytokine production (Figure 2) (62–64). After antigen binding by the B cell receptor (BCR), the antigen is internalized, processed, and expressed on the surface of the B cells as a complex with major histocompatibility complex (MHC)-I or II molecules. Additional to antigen-presentation molecules, costimulatory molecules, such as CD80, CD86, and CD40, are expressed on B cells and contribute to optimal T cell activation (65). Myelin reactive peripheral B cells can induce CD4^+^ T cell responses in a proportion of MS patients (66). Additionally, B cell expression of the costimulatory molecules CD80 and CD86 is higher in MS patients than healthy controls (67, 68).

Finally, B cells support or regulate effector immune functions via the secretion of different cytokines (Figure 2). B cell activation factor (BAFF) and A Proliferation-Inducing Ligand (APRIL) are important survival factors for B cells and plasma cells, thereby maintaining the B cell pool (69). BAFF expression is upregulated in active and inactive MS lesions (70, 71). Maintaining BAFF expression within certain limits in order to balance pro-inflammatory and regulatory B cell subtypes can be an important feature for MS therapies. B cells support pro-inflammatory functions through secretion of tumor necrosis factor alpha (TNF-α), interleukin (IL)-6, and lymphotoxin alpha (LT-α) and exert regulatory functions via the production of IL-10 and IL-35 (22, 23, 72–75). In healthy individuals, transitional B cells perform regulatory functions by producing IL-10, thereby suppressing antigen-mediated T cell activity (26). Within the CD27^+^ memory B cell and plasma cell population, IL-10 and IL-35 producing Bregs can be enriched, showing that more mature B cells can also have regulatory functions next to antibody production and T cell activation (23, 25, 76–78). B cells from MS patients showed an increased production of IL-6, an increased LT-α/IL-10 ratio and increased LT-α and TNF-α production after stimulation *in vitro* (70). In addition, B cells from untreated MS patients secreted more pro-inflammatory IL-6 and less regulatory IL-10 than those from healthy controls (37, 79, 80).

### Additional Proof of B Cell Involvement in MS

Additional proof of B cell involvement in MS came from analysis of BCR sequences and genetic and animal studies. Analysis of Ig heavy chain variable sequences (VH) of intrathecal B cells from MS patients showed a restricted usage of Ig VH gene segments, pointing to a chronic antigen-driven B cell response in MS patients (81–83). Genetic studies in MS identified susceptibility genes that show a strong association with B cell function, such as HLA-DRB1*1501, HLA-DRB5*0101, and HLA-DQB1*0602 (84). Also observations from clinical trials of the B cell-depleting anti-CD20 monoclonal antibody rituximab indicated the importance of antibody-independent B cell functions in the pathogenesis of MS. These clinical studies showed an unchanged level of total Ig and a decrease in CSF T cell numbers, providing additional proof that B cells highly interact with T cells in MS (85–87).

Other information about the involvement of B cells in the pathogenesis of MS is available from experimental autoimmune encephalomyelitis (EAE), the animal model of MS. The role of B cells in EAE has long-time been neglected as B cells are not essential contributors to EAE models based on peptide immunization. More recent studies using recombinant MOG protein immunization have highlighted the role of B cells in EAE induction and pathology (88). The dual role of B cells in EAE was indicated by the use of anti-CD20 treatment, as disease exacerbation was evident when depleting Bregs before EAE induction while disease severity decreased when depleting memory B cells after EAE induction (22, 89, 90). B cells were essential for the generation of optimal pathogenic CD4^+^ T cell responses and differentiation of MOG specific T-helper (Th)1 and Th17 cells (91). In B cell deficient mice, EAE induction by adoptive transfer of activated T cells was reduced and reactivation of infiltrated T cells was impaired (92). Further, B cell-specific MHC class II-deficient mice were resistant to EAE induction and exhibited diminished Th1 and Th17 responses (93). Hence, B cells can promote EAE induction by acting as antigen-presenting cells. Moreover, B cell antigen presentation was proven to be crucial for maximal disease in EAE, further emphasizing the importance of B cells in MS pathogenesis (94).

Recently, a direct link between peripheral and intrathecal B cells was demonstrated. Clonally expanded autoreactive B cells with signs of affinity maturation were, next to the CSF, found in the PB of MS patients (82, 95). Further, expanded B cell clones were found both in the PB/draining cervical lymph nodes and the CSF, indicating a complex crosstalk between the periphery and the CNS in MS pathogenesis (27, 96). Exchange of B cells between the CSF and the PB may suggest that B cells carry antigen from the CNS to peripheral secondary lymphoid organs (11). Primed T cells then migrate to the CNS where residing B cells may further promote T cell activation. These data underline the importance of using therapeutics based on the inhibition of B cell transmigration into the CNS or that induce peripheral B cell depletion (11, 27, 96, 97). Additionally, autoreactive B cells can be removed from the B cell pool via both a central and a peripheral checkpoint. It seems that especially the peripheral tolerance checkpoint is defective, as shown by the equal proportion of polyreactive and anti-nuclear transitional B cells in MS patients and healthy donors (normal central B cell tolerance) and the increased proportion of mature naive B cells from MS patients reactive toward peripheral and CNS self antigens (defective peripheral B cell tolerance) (98). This defect is probably due to impaired Treg function that leads to the accumulation of autoreactive B cells (99). All these observations strengthen the idea that PB B cells contribute to the pathogenic B cell pool present in the CNS of MS patients and are involved in MS pathogenesis both by antibody-dependent and -independent B cell functions. Thus, investigating PB B cells and the effects of treatment on peripheral B cell functions may contribute to our understanding of the pathogenesis of MS (80, 85–87).

This review summarizes how current MS treatments influence B cell functions. At the moment, numerous FDA approved MS treatments or drugs in clinical trials can be subdivided in first-, second- and third-line therapies (Tables 1–3). Generally established first-line therapies include interferon-beta (IFN-β) and glatiramer acetate (GA), while fingolimod and natalizumab are considered to be second-line treatments. The recently approved oral drugs teriflunomide and dimethyl fumarate (DMF) are oral treatments used as first-line treatment for MS (100–105). Second- and third-line antibody treatments are rituximab, alemtuzumab, ocrelizumab, ofatumumab, and antibodies that target BAFF and APRIL. Modulating B cell functions is an important tool for treating MS patients, although information on the effects of therapy on B cell functions is limited. Investigating the effects of treatment on B cell functions is of potential relevance to the efficacy of such treatments and it will help to increase our insight into the involvement of PB B cells in MS pathogenesis.

**Table 1:** **Overview of first-line MS treatments**.

Name	Target	Primary mode of action	MS type	Important clinical observations	Reference
IFN-β1a *Avonex*^®^, IFN-β1a *Rebif*^®^, IFN-β1b *Betaferon*^®^	/	• Increases the expression of anti-inflammatory agents while downregulating pro-inflammatory cytokines	RRMS	• Reduction in relapse rate, magnetic resonance imaging (MRI) lesion activity, brain atrophy, risk of sustained disability progression	(100, 107, 152–157)
		• Shifts the immune response from a T-helper (Th) 1 phenotype to Th2		• Increase in time to reach clinically definite MS after the onset of neurological symptoms	
		• Reduces trafficking of inflammatory cells toward the BBB			
Glatiramer acetate *Copaxone*^®^	/	Induces tolerogenic T cell immune responses and CD4^+^ and CD8^+^ regulatory T cells due to mimicry of MBP	RRMS	• Reduction in relapse rate	(79, 105, 158–162)
				• Improvement of disability measured using Expanded Disability Status Scale (EDSS)	
Teriflunomide *Aubagio*^®^	Dihydroorotate dehydrogenase	Inhibits *de novo* pyrimidine synthesis by blocking the enzyme dihydroorotate dehydrogenase	RRMS	• Reduction in exacerbation rate, annualized relapse rate, risk of sustained accumulation of disability	(163–168)
Dimethyl fumarate, BG-12 *Tecfidera*^®^	/	• Interferes in the citric acid cycle	RRMS	• Reduction in annual relapse rate	(102, 108, 109, 168)
		• Activates the nuclear factor (erythroid-derived 2)-like 2 (Nrf2) pathway		• Reduction in disability progression	

**Table 2:** **Overview of second-line MS treatments**.

Name	Target	Primary mode of action	MS type	Important clinical observations	References
Natalizumab *Tysabri*^®^	VLA-4 (α4-integrin)	Inhibits migration of lymphocytes to the CNS	RRMS	• Reduction in exacerbation rate, annual relapse rate and disability rate	(169–171)
FTY720 *Fingolimod*^®^	Sphingosine-1-phosphate receptor (S1PR)	• Downregulates S1PR on lymphocytes	RRMS	• Reduction in relapse rate, disability progression and total number of gadolinium-enhancing lesions	(172–180)
		• Inhibits egression from lymphoid organs into the circulation			

**Table 3:** **Overview of third-line MS treatments**.

Name	Target	Primary mode of action	MS type	Important clinical observations	References
Monoclonal anti-CD20 antibody rituximab *rituxan*^®^, *mabThera*^®^, *zytux*^®^	CD20	Depletes CD20^+^ B cells	RRMS	• Reduction of new brain lesions and clinical relapses	(85, 87, 181–185)
			PPMS		
			SPMS		
Monoclonal anti-CD20 antibody *ocrelizumab*^®^	CD20	Depletes CD20^+^ B cells	RRMS	• Reduction in gadolinium-enhancing (Gd) T1 lesions, in total number of new and persisting Gd-enhancing lesions and in annualized relapse rate	(181, 186, 187)
			PPMS	• Improved efficacy compared with rituximab with lesser infusion-related reactions	
Monoclonal anti-CD20 antibody *ofatumumab*^®^	CD20	Depletes CD20^+^ B cells	/	• Reduction in cumulative number of new Gd-enhancing lesions and new and enlarging T2 lesions	(188–191)
Alemtuzumab *campath*^®^*, lemtrada*^®^	CD52	Depletes CD52^+^ B and T cells	RRMS	• Reduction in rate of sustained accumulation of disability, disability progression, and the annualized rate of relapse	(120, 192–194)
				• Improvement of disability scores	
Anti-BAFF; anti-APRIL *atacicept*^®^, belimumab *benlysta*^®^, tabalumab, blisibimod	BAFF and/or APRIL	Blocks activation of B cells via inhibition of BAFF and APRIL or the BAFF receptor	RRMS	• Increase in inflammatory disease activity and annualized relapse rate (atacicept^®^)	(70, 149–151, 195, 196)

## Effects of Treatment on Total B Cell Numbers

**Figure 3:**
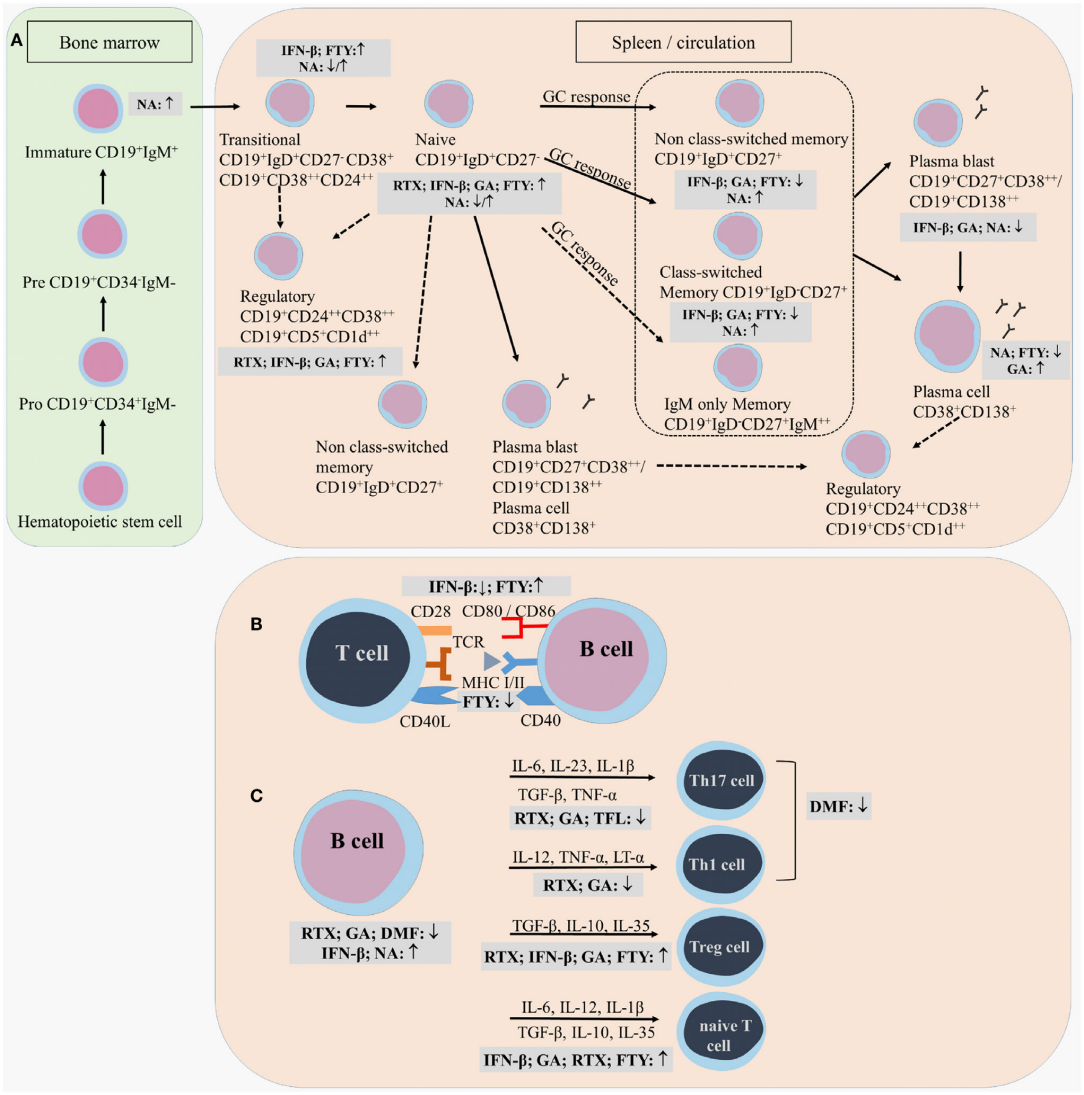
**Effects of immunomodulatory therapy on B cell subtype distribution and function**. B cell development in the bone marrow and periphery **(A)**, antigen presentation and costimulatory molecules expressed on the B cell surface **(B)** and B cell cytokine production **(C)** are shown together with the effects of treatment on the different B cell subtypes and functions. CD, cluster of differentiation; IFN-β, interferon-β; FTY, fingolimod; GA, glatiramer acetate; NA, natalizumab; DMF, dimethyl fumarate; TFL, teriflunomide; RTX, rituximab; IL, interleukin; TGF, transforming growth factor; TNF, tumor necrosis factor; Th, T helper.

Total B cell numbers and percentages in the PB were changed during treatment, both in cross-sectional and longitudinal studies, with an increase in the frequency of CD19^+^ B cells in IFN-β-treated MS patients and a decrease in GA- and DMF-treated MS patients (Figure 3) (79, 106–110). Different studies indicated that the percentage of B cells was increased in the PB and decreased in the CSF of natalizumab-treated MS patients, due to the inhibition of lymphocyte migration into the CNS (106, 111–118). This increase in PB B cells was observed up to 30 months after start of the treatment (112). Opposite effects were observed in fingolimod-treated MS patients where total B cell numbers in the PB were diminished because of the lymphocyte entrapment within secondary lymphoid organs. No changes were observed in CSF B cell numbers under fingolimod treatment (118, 119). In a study with 69 RRMS patients treated with rituximab, a decrease of 95% in the percentage of CD20^+^ B cells was evidenced from 2 weeks after treatment until 24 weeks (87). By week 48, B cells returned to 31% of baseline values. Alemtuzumab treatment caused a general depletion of both T and B cells in the PB of treated patients (120).

## Effects of Treatment on B Cell Subtype Distribution

Different effects on B cell subtype distribution were demonstrated using different MS treatments. An increased frequency of immature and transitional B cells was generally evidenced under different treatments, including IFN-β, natalizumab, fingolimod, and during repopulation following rituximab or alemtuzumab treatment (106, 107, 111, 113, 120–124). These reports all point toward an increased output of B cells from the bone marrow under immunomodulatory treatment. In this regard, an increased release of lymphoid committed progenitor cells was shown during natalizumab therapy in MS (112). However, in a cross-sectional study with 8 natalizumab-treated RRMS patients, a significant decrease in the percentage of transitional B cells was evidenced (106). Also in fingolimod treated MS patients, the output of newly produced B cells or immature B cells from the bone marrow was decreased (121).

Therapeutic effects on frequencies of Bregs have only been described in relation to the use of fingolimod, rituximab and alemtuzumab therapy. In 48 fingolimod treated MS patients, a proportional increase of Bregs was recently described compared to 74 untreated MS patients and 70 healthy controls (125). During repopulation after B cell depletion by rituximab or alemtuzumab, naive B cells with an increased expression of CD38 and CD5, which are described as Bregs, were predominantly present, both in MS and other autoimmune diseases (31, 122, 126).

Peripheral blood naive B cells were increased in IFN-β, GA, natalizumab, and fingolimod-treated MS patients in comparison with treatment-naive MS patients in different cross-sectional and longitudinal studies (79, 106, 115, 119, 127). This indicates that the B cell population shifts toward a less disease promoting B cell pool after different MS treatments. For GA and natalizumab, this could not be reproduced in other studies where a decreased frequency of naive B cells was observed or no change in B cell subtype distribution at all (106, 107, 127). However, no information was available about the treatment duration, which makes it difficult to compare the studies.

Also contributing to a less disease promoting B cell phenotype is the significant decrease in the frequency of non-class-switched, class-switched memory B cells, and plasma blasts in both cross-sectional and longitudinal studies of IFN-β-, GA-, and fingolimod-treated MS patients, even when using different B cell classifications (36, 79, 106, 107, 119, 125, 127–129). Although a decrease in the proportion of plasma blasts was observed in natalizumab-treated MS patients, a higher percentage of memory and marginal zone B cells was reported (112, 114, 115, 127, 130). This memory B cell increase is probably due to the reduced retention of memory B cells in the spleen (112). In the CSF, natalizumab treatment particularly depleted CD5^+^ B cells and plasma blasts (131).

Data on B cell subtype distribution are missing for DMF and teriflunomide-treated MS patients. *In vitro* studies have shown that teriflunomide induces cell cycle arrest in B cells without inducing apoptotic cell death (101, 132, 133). Moreover, the effects of different third-line treatments on B cell subtype distribution is poorly investigated in MS as not all treatments are FDA approved and clinical trials are ongoing. To our knowledge, no data are available on the repopulation of B cells after discontinuation of the B cell-depleting therapies ocrelizumab and ofatumumab. Further research is warranted to increase the understanding of the exact mechanism of action and to investigate restoration of the immune balance following depletion therapies.

From this overview, we can conclude that immunomodulatory treatment of MS patients induces a shift in the distribution of B cell subtypes toward a more regulatory or anti-inflammatory phenotype. This is of high clinical importance as a disturbed balance between the different B cell subtypes is observed in MS. For different MS treatments, the effects on B cell subtype distribution have already been investigated to some extent, still conflicting data are present. This is probably due to variation in measurement time points and B cell characterization strategies. Furthermore, as each treatment requires a different time to reach a steady state of immunological parameters and treatment efficiency, it is difficult to compare study results. Therefore, it is essential to use a longitudinal design of the study and take into account the pharmacodynamical properties of the treatment, since some treatment effects could get lost when only measuring in a cross-sectional manner. B cell subtype analysis can also be highly relevant in the search for new markers for progressive multifocal leukoencephalopathy (PML) in natalizumab-treated patients, as B cells were described as potential carriers of the John Cunningham (JC) virus into the CNS (134). Other research is focused on finding risk factors for the development of PML during natalizumab treatment (112, 130, 135, 136).

## Effects of Treatment on B Cell Effector Function

Here, we present the available data on the effect of immunomodulatory treatment on antibody-dependent and -independent B cell functions. These include antibody production, antigen presentation, costimulation, migration, and cytokine production (Figure 3).

### Effects of Treatment on Antibody Production

Glatiramer acetate treatment did not change serum levels of total IgG and IgM in MS patients, but *in vitro* levels of IgG and IgM antibodies were increased after stimulation of PB B cells from these patients (79). Natalizumab-treated MS patients showed lower levels of IgM in both serum and CSF and lower anti-neurofilament light antibodies in the serum than non-natalizumab-treated MS patients (116, 137). Longitudinal data of 24 MS patients confirmed these results with a decrease in neurofilament light antibody levels, a decline in total IgG levels in the PB and CSF, and a decline in total IgM in the PB (116, 137). Further, the IgG index, which reflects intrathecal IgG production, was decreased during natalizumab treatment, resulting in the disappearance of OCB in some of the treated MS patients (138). Whether a decline in the anti-neurofilament light antibodies is a consequence of a decrease in total antibody levels is not stated. Additionally, vaccination studies in fingolimod-treated healthy volunteers have demonstrated a mild to moderate decrease in IgG and IgM antibody levels toward some antigens, suggesting that fingolimod could reduce autoantibody production in MS as well (139). Teriflunomide, in contrast, did not influence immune responses toward influenza vaccines, indicating that the protective immune responses are preserved in these patients (140).

The anti-BAFF antibody atacicept^®^ did not show beneficial results in clinical trials for MS and even led to worsening of the disease. More patients with optic neuritis who received atacicept^®^ progressed to clinical definite MS (141). The efficacy of this therapy was proven in a clinical trial for RA wherein circulating IgG and IgA rheumatoid factor (RF) and total IgM, IgA, and IgG levels were reduced (142, 143). These observations indicate that, although MS and RA are both autoimmune diseases in which B cells are involved, different effector mechanisms of B cells are involved in both diseases. Since atacicept^®^ affects antibody-producing plasma cells and clinical efficacy of atacicept^®^ is shown in RA, one can speculate that in RA pathogenesis autoantibody production is more important than in MS pathogenesis. This underlines the multifactorial functions of B cells in autoimmunity.

### Effects of Treatment on B Cell Antigen Presentation, Costimulation, Migration

Most information on effects of treatment on B cell antigen presentation, costimulation, and migration is available for IFN-β. *Ex vivo* analysis of PB B cells from 15 IFN-β-treated MS patients showed a decreased percentage of CD80, CD86, and CCR5 positive total and CD27^−^ naive B cells compared to untreated MS patients (36). This pointed toward a less migratory and costimulatory phenotype of these B cells in the PB under treatment, which was confirmed *in vitro* (36, 144, 145). Furthermore, the increase in CD80 positive cells during relapses in MS patients was shown to be counteracted by IFN-β treatment (68). Since CD80 expression is associated with a Th1 phenotype and CD86 expression is associated with a Th2 response, these findings could indicate a shift from Th1 to Th2 in IFN-β treated MS patients (146). Within the CD27^+^ memory B cell compartment, the percentage of CD86 positive B cells was increased while the percentage of CXCR3 positive cells was decreased in the IFN-β group compared to healthy controls, indicating that memory B cells were less able to migrate to the CNS (36). IFN-β pretreated B cells were less able to induce proliferation of anti-CD3 and anti-CD28 stimulated CD4^+^ T cells than untreated B cells, further proving the immunomodulatory capacity of IFN-β therapy (144).

In a longitudinal study, B cell expression of the adhesion marker intracellular adhesion molecule (ICAM)-3 was reduced during GA treatment, indicating a potential role for GA in controlling the migration of B cells toward the CNS (147). Other longitudinal data showed a decrease in B cell expression of the antigen-presenting molecule human leukocyte antigen (HLA)-DR/DP/DQ and an increase in CD80 and CD86 costimulatory molecules on PB B cells in fingolimod treated MS patients (119). In contrast, a decreased expression of CD80 and stable CD86 expression was evidenced on B cells from fingolimod treated MS patients when compared to untreated MS patients in another study (128).

No data are present, to our knowledge, concerning the effects of DMF, teriflunomide, natalizumab and the CD20-depleting antibodies like rituximab, ocrelizumab, and ofatumumab on B cell surface expression of antigen presentation, costimulation, and migration markers. Natalizumab treatment could indirectly have an effect on these B cell functions due to the observed B cell subtype redistribution and general immune modulation. Because DMF and teriflunomide are recent FDA approved drugs, more research is warranted to investigate the effects of these treatments on B cell functions. Still, it can be concluded that different MS therapies can influence the interaction of B cells with T cells or other immune cells. As a consequence, inflammatory responses that are detrimental for the CNS are tempered, which is reflected in the clinical outcome of the treated MS patients.

### Effects of Treatment on Cytokine Production by B Cells

In a cross-sectional study of IFN-β treated RRMS patients, increased serum levels of BAFF were observed compared to healthy controls, untreated, and GA-treated RRMS patients (107, 148). Twelve months after discontinuation of alemtuzumab treatment, increased serum BAFF levels were also observed (122). The BAFF-depleting antibody atacicept^®^ exacerbated MS, which could be due to the decreased functionality of Bregs, as BAFF and APRIL signaling is highly implicated in the survival of Bregs. Still, the exact reason for the observed increased disease activity needs to be elucidated (149–151).

In terms of changes in cytokine production, IFN-β treatment caused induction of IL-10 production by B cells *in vitro* (144). Although GA did not directly modulate B cell proliferation or cytokine secretion *in vitro* (9), *ex vivo* analysis showed an increased secretion of IL-10 by B cells of 22 RRMS patients treated with GA (79). Intracellular flow cytometric analysis of B cells isolated from GA treated MS patients showed no increased frequency of IL-10 positive B cells in the PB of MS patients, indicating that GA does not influence the number of cytokine producing cells but rather the secretion of the cytokines (79). Further, a decreased capacity to secrete LT-α and IL-6 was indicated after B cell stimulation via CD40 and CD40L interaction or via Toll-like receptor triggering (79). An elevated IL-10 production was also evidenced for PB B cells from fingolimod-treated MS patients and repopulated B cells after rituximab treatment (31, 80, 125, 128). In fingolimod treated MS patients, the increased IL-10 production was accompanied by a decreased TNF-α production, while B cells following rituximab treatment secreted less pro-inflammatory cytokines IL-6, LT-α, and TNF-α (31, 80, 125, 128). Limited data is present of the effects of DMF and teriflunomide on the immune function in MS patients. In psoriasis patients, it was shown that DMF altered the immune and T cell cytokine profile (102, 110). Teriflunomide limits the secretion of pro-inflammatory molecules by immune cells, including IL-6 and IL-8 (101).

Thus, similar effects have been observed for all studied treatments on the cytokine production by B cells, correcting the imbalance between regulatory and disease promoting B cell functions in MS. We have to keep in mind that since different B cell subtypes produce different cytokines, by changing B cell subtype distribution, cytokine balances are changed as a secondary effect of the treatment. Data are missing on the effects of treatment on cytokine production by B cells for some FDA approved treatments such as natalizumab and for some treatments in clinical trials such as anti-CD20 monoclonal antibodies. It can be speculated that a potential mode of action by which these treatments contribute to the improvement of MS pathogenesis can be by influencing B cell cytokine production from a pro-inflammatory phenotype toward a more regulatory phenotype, still this needs to be further investigated.

## Conclusion

It is eminent that B cells are major players in MS pathogenesis and contribute to the disease via both antibody-dependent and -independent mechanisms. B cells are essential for antigen presentation and costimulation of T cells, for the production of cytokines and to produce antibodies that will target components of the CNS. Thus, focusing on effects of treatment on these cells will help in our understanding of MS pathogenesis. Although initially not designed for that purpose, many MS modifying treatments influence both antibody-dependent and -independent B cell functions. Research on effects of therapy on B cell phenotype and function has demonstrated a shift from pro-inflammatory B cell functions toward more anti-inflammatory and regulatory functions. Still, each treatment influences this balance in its own manner. IFN-β, natalizumab, fingolimod, BAFF and APRIL targeting monoclonal antibodies, rituximab and alemtuzumab, induce compositional changes of the B cells, resulting in a less disease promoting distribution of B cells in both the PB and CSF of MS patients. GA, DMF, and teriflunomide work primarily via modulating B cell cytokine production. Still, all these effector mechanisms of B cells are interconnected and cannot be separated from each other. Investigating the mechanism of action of different treatments in different autoimmune diseases leads to new insights into that specific disease. For example, atacicept^®^ has different clinical effects in RA and MS, indicating different roles of B cells in these diseases. More research is needed since inconsistencies between studies are present due to differences in B cell subtype definition and time point of measurement. Consensus in B cell subtype characterization will have added value in future research. Further, researchers should take into account pharmacodynamics of the compounds in order to decide on the specific time point for measuring B cell characteristics. Analysis of treatment effects on B cell subtype distribution and function can alternatively lead to prognostic knowledge for determining therapy efficiency. Finally, research should focus on finding specific therapies for the treatment of SPMS although initial efforts have been made. Further analysis of B cell functions in MS pathogenesis and the effects of treatment on these functions is hereby important in order to increase insight into the role of B cells in the disease process. This could lead to the development of novel and more specific therapies.

## Conflict of Interest Statement

The authors declare that the research was conducted in the absence of any commercial or financial relationships that could be construed as a potential conflict of interest.
